# Possible association between common variants of the phenylalanine hydroxylase (*PAH*) gene and memory performance in healthy adults

**DOI:** 10.1186/1744-9081-9-30

**Published:** 2013-07-30

**Authors:** Toshiya Teraishi, Daimei Sasayama, Hiroaki Hori, Noriko Yamamoto, Takashi Fujii, Junko Matsuo, Anna Nagashima, Yukiko Kinoshita, Kotaro Hattori, Miho Ota, Sayaka Fujii, Hiroshi Kunugi

**Affiliations:** 1 Department of Mental Disorder Research, National Institute of Neuroscience, National Center of Neurology and Psychiatry, 4-1-1, Ogawahigashi, Kodaira, Tokyo 187-8502, Japan

**Keywords:** Genetic polymorphism, Phenylalanine hydroxylase, Verbal memory, Wechsler Memory Scale-Revised (WMS-R), Association study

## Abstract

**Background:**

Phenylalanine hydroxylase (PAH) is the enzyme that metabolizes phenylalanine, an essential amino acid required for catecholamine synthesis. Rare mutations in *PAH* are causal to phenylketonuria (PKU), an autosomal recessive disease characterized by neuropsychiatric symptoms including intellectual disability. We examined whether there is an association between common single nucleotide polymorphisms (SNPs) of *PAH* and memory performance in the Japanese population.

**Methods:**

Subjects were 599 healthy adults (166 males and 433 females; mean age 43.8 ± 15.5 years). The Wechsler Memory Scale-Revised (WMS-R) was administered to all participants to assess memory performance. Genotyping was performed for 6 selected tagging SNPs of *PAH* (rs1722387, rs3817446, rs1718301, rs2037639, rs10860936 and rs11111419).

**Results:**

Analyses of covariance controlling for sex and education years, indicated a significant association between a SNP (rs2037639) and age-corrected verbal memory index of WMS-R (nominal p = 0.0013) which remained significant after correction for multiple testing ( p = 0.0013 < 0.0017 = 0.05/30tests). Individuals with the GG genotype showed a significantly lower mean verbal memory score, compared with those individuals carrying the AA/AG genotype (106.0 ± 16.0 vs. 111.7 ± 13.4; p = 0.00099). A haplotype block containing two markers of rs2037639 and rs10860936 was associated with verbal memory index (permutation global p = 0.0091).

**Conclusions:**

Our findings suggest that common genetic variations in *PAH* are associated with verbal memory in healthy adults. Unknown functional polymorphisms in *PAH* or those in other genes nearby might affect memory performance.

## Background

Accumulating evidence suggests that genetic factors influence human complex behavior, including neurocognition throughout the life span [1,2]. Studies of single genes that cause monogenic diseases with cognitive impairments will advance understanding of genetic and molecular mechanisms underlying individual differences in neurocognitive performance [3]. Phenylketonuria [PKU; McKusick OMIM 261600], an inborn error of phenylalanine metabolism, causes intellectual disability (i.e., developmental disorder with general cognitive disabilities), seizures, microcephaly, motor disorder, skin rashes and other symptoms, including psychiatric conditions such as depression [4-6]. About 98% of PKU is caused by mutations in *phenylalanine hydroxylase* (*PAH*) on chromosome 12q. *PAH* encodes phenylalanine hydroxylase [EC 1.14.16.1] that converts phenylalanine to tyrosine which is a precursor of the neurotransmitter dopamine and other catecholamines [5,6]. More than 500 mutations in *PAH* were registered as responsible for PKU (http://www.pahdb.mcgill.ca/) [6].

Interestingly, previous studies [7,8] reported that polymorphisms in *PAH* confer susceptibility to schizophrenia, a disease characterized by pervasive neurocognitive deficiencies including distinct memory impairment [9], as well as with psychiatric symptoms.

Based on these observations, we hypothesized that common genetic variations in *PAH* might be associated with neurocognitive function, memory performance in particular. The aim of the present study was to examine the possible association of single nucleotide polymorphisms (SNPs) of *PAH* with memory performance in healthy adults. To our knowledge, there has been no report that examined such an association.

## Methods

### Subjects

Subjects were 599 healthy Japanese adults (166 males and 433 females; mean age 43.8, standard deviation [SD] 15.5 years). Mean education years of the participants were 15.2 ± 2.7 years. All subjects were biologically unrelated and were recruited via advertisements of free local magazines in the western part of Tokyo Metropolitan and our website announcement. All subjects underwent structured interview using the Japanese version of the Mini International Neuropsychiatric Interview (M.I.N.I.) [10,11] and additional unstructured interview by a research psychiatrist to confirm no current or past history of any axis І or II disorders in the Diagnostic and Statistical Manual of Mental Disorders, 4th edition (DSM- IV) criteria [12]. Participants were excluded if they had a current or past contact to psychiatric services, or had a history of severe head injury, serious central nervous system or physical disease, regular use of psychotropics, or substance abuse/dependence.

### Assessment of memory performance

The Japanese version of Wechsler Memory Scale-Revised (WMS-R) [13,14] was administered to all participants [15,16]. Raw scores of 5 indices, ie, the verbal, visual and general memory, attention/concentration, and delayed recall calculated from 13 subtests of WMS-R were converted to age-corrected standard scores according to the test manual [14]. The study was approved by the ethics committee at the National Center of Neurology and Psychiatry (NCNP), Japan and was performed in accordance with the Declaration of Helsinki. Every subject gave written informed consent after full explanation of the study aim and protocol.

### SNPs-selection and genetic analysis

Preparation of genomic DNA from venous whole blood of subjects was performed according to standard procedures. We selected 6 tagging SNPs (rs1722387, rs3817446, rs1718301, rs2037639, rs10860936 and rs11111419) throughout *PAH*, encompassing 2 kilobase (kb) of sequence upstream and downstream (between chromosome 12 positions 101,754,235 and 101,837,511), using the ‘Tagger’ application of the program Haploview 4.2 (http://www.broad.mit.edu/mpg/haploview/) (version 3.0 release R2) [17]. We used the HapMap genotype data of the Han Chinese from Beijing (CHB) and the Japanese from Tokyo (JPT) and an r^2^ threshold of 0.8 with a minor allele frequency (MAF) threshold of 0.1. Genotyping of the SNPs was performed using the TaqMan allelic discrimination assay (Applied Biosystems, Foster City, CA, USA) with 10 ng human DNA and 1.5 μl 2× qPCRTM Mastermix Plus Quick Gold Star (EUROGENTIC, Seraing, Belgium). Polymerase chain reaction was performed with an initial cycle at 95°C for 10 minutes followed by 50 cycles of 92°C for 15 seconds and 60°C for 1 minute in GeneAmp PCR System 9700 (Applied Biosystems). The allele-specific fluorescence was discriminated with an ABI PRISM 7900 Sequence Detection Systems (Applied Biosystems). Genotype data were determined blind to the WMS-R scores. Ambiguous genotype data were excluded from the analysis. The call rate of non-missing genotypes was over 97%.

### Statistical analysis

The statistical package for the social sciences (SPSS) version 11.0 (SPSS Japan, Tokyo) was used for the analysis of variance (ANOVA), analysis of covariance (ANCOVA) and χ^2^ test. Demographic characteristics between genotype groups was compared by using ANOVA or χ^2^ test, as appropriate. The ANCOVA was performed to test the effect of genotype on each score of the 5 indices of WMS-R, controlling for sex and education years. Paired comparisons for verbal memory scores of each genotype were carried out with the ANCOVA controlling for sex and education years. Haploview 4.2 was used to check deviation from the Hardy-Weinberg equilibrium (HWE) for each SNP, and to calculate measures of linkage disequilibrium (LD). Haplotype blocks were constructed using the default block search algorithm of Gabriel *et al.*[18]. Haplotypes with frequencies below 1% were not included in the association analysis. The PLINK statistical package version 1.07 (http://pngu.mgh.harvard.edu/~purcell/plink) [19] was used for haplotype analyses with sex and education years, and empirical p values were obtained based on 10,000 permutations. For the power calculation, G*power software version 3.1.5 (http://www.psycho.uni-duesseldorf.de/abteilungen/aap/gpower3/) was used [20]. Statistical tests were two-tailed and p values < 0.05 were considered significant. Since, as to the association analyses between 6 SNPs and 5 indices of WMS-R, we conducted 30 comparisons, critical p value for multiple testing was set at 0.0017 (=0.05/30 tests). Continuous variables were described as mean ± SD.

## Results

**Figure 1:**
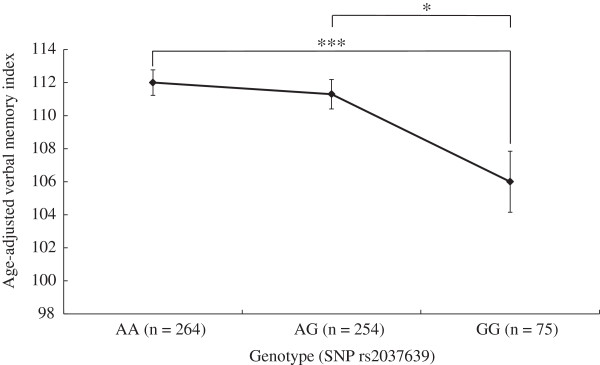
**Mean age-adjusted verbal memory scores of WMS-R grouped by genotypes of *PAH *SNP rs2037639.** Data are expressed as mean ± standard error. *p < 0.05, ***p < 0.001: significant differences by analysis of covariance controlling for sex and education years.

There were no significant group differences in age or smoking status between the genotype groups (e.g., rs2037639 AA/AG/GG) (Additional file 1: Table S1). With respect to education years, a nominally significant group difference between the genotype groups was observed for rs2037639 (Additional file 1: Table S1). Therefore in subsequent analyses, sex and education years were controlled for. Characteristics of the 6 tag SNPs are shown in Table 1. No significant deviation from the HWE was found for the selected 6 markers. Table 2 shows mean scores of the 5 indices of WMS-R and nominal p values obtained by ANCOVA, in which memory index was a dependent variable, genotypes and sex were independent variables and education years was a covariate. We found 5 out of 30 tests (6 SNPs × 5 indices of WMS-R) were nominally significant without correction for multiple testing. Interestingly, all 5 tests were associations with verbal memory index. Even after Bonferroni correction for multiple testing, the association between rs2037639 and verbal memory index remained significant (p = 0.0013 <0.0017 = 0.05/30). In addition, we compared indices of WMS-R in only nonsmokers to avoid the possible confounding effects of smoking. ANCOVA controlling for sex and education years showed that the association between rs2037639 and age-corrected verbal memory index of WMS-R was significant for multiple testing (p = 0.00024 < 0.0017, F = 8.5). Therefore, we do not think that the association between rs2037639 and age-corrected indices of WMS-R was attributable to smoking status. In subsequent analyses, we focused on the effect of rs2037639 on verbal memory. Figure 1 shows mean verbal memory scores depending on genotypes of rs2037639. There was a significant difference in mean verbal memory score across the three genotype groups (F = 6.7, df = 2, p = 0.0013) (AA genotype: 112.0 ± 12.6, n = 264; AG: 111.3 ± 14.2, n = 254; GG: 106.0 ± 16.0, n = 75). In paired comparisons, subjects with GG genotype showed a significantly lower mean verbal memory score, compared with subjects with AG (F = 5.4, df = 1, p = 0.021) and AA genotype (F = 13.4, df = 1, p = 0.00029). No significant difference was observed in mean verbal memory score between individuals with AA and AG genotype (data not shown). When the AG and AA genotype groups were combined, ANCOVA controlling for sex and education years showed that the GG genotype group was significantly lower in mean verbal memory score than the AA/AG group (106.0 ± 16.0 vs. 111.7 ± 13.4; F = 10.9, df = 1, p = 0.00099).

**Table 1:** **Characteristics of selected 6 tag SNPs of *PAH***

**SNP**	**Chromosome location**	**Location within *PAH***	**HWE p value**	**Call rate (%)**	**SNP alleles**	**Minor allele**	**MAF**
rs1722387	101765200	Intron 8	0.60	98.2	A/G	A	0.143
rs3817446	101784949	Intron 4	0.21	99.2	A/G	A	0.242
rs1718301	101795323	Intron 4	0.64	97.3	A/G	A	0.166
rs2037639	101795480	Intron 3	0.26	98.8	A/G	G	0.341
rs10860936	101807082	Intron 3	0.77	98.2	C/T	C	0.122
rs11111419	101809173	Intron 3	0.66	98.8	A/T	T	0.223

*HWE* Hardy-Weinberg Equilibrium, *MAF* minor allele frequency.

**Table 2:** **Association between *PAH *polymorphisms and indices of the WMS-R**

**WMS-R Index**	**Mean ± S.D.**	**SNP of *PAH *gene**																	
		**rs1722387**			**rs3817446**			**rs1718301**			**rs2037639**			**rs10860936**			**rs11111419**		
Genotype		A/A	A/G	G/G	A/A	A/G	G/G	A/A	A/G	G/G	A/A	A/G	G/G	C/C	C/T	T/T	A/A	A/T	T/T
Verbal memory	110.7 ± 14.0	104.6	109.6	111.2	104.8	111.9	111.0	113.5	110.1	110.9	112.0	111.3	106.0	104.8	108.9	111.4	111.6	110.5	103.9
		F = 3.57, **p =0.029**			F = 3.8, **p =0.023**			F = 2.1, p =0.12			F = 6.7, **p =0.0013**			F = 5.5, **p =0.0042**			F = 5.0, **p =0.0067**		
Visual memory	107.6 ± 11.2	106.4	107.9	107.6	107.1	108.2	107.3	110.0	108.1	107.5	107.5	108.3	107.0	107.5	107.7	107.7	107.9	107.0	109.1
		F = 0.26, p =0.77			F = 0.041, p =0.96			F = 0.80, p =0.45			F = 0.13, p =0.88			F = 0.33, p =0.72			F = 1.4, p =0.26		
General memory	111.3 ± 12.8	105.8	111.3	111.5	107.0	112.7	111.2	114.5	110.9	111.5	112.0	112.1	108.1	106.2	110.8	111.7	111.9	111.2	107.4
		F = 0.89, p =0.41			F = 1.9, p =0.15			F = 2.4, p =0.089			F = 2.8, p =0.060			F = 1.5, p =0.22			F = 1.4, p =0.26		
Attention/Concentration	106.3 ± 13.4	104.1	106.9	106.2	106.0	106.8	106.2	103.2	106.8	106.1	107.0	106.0	105.6	107.1	106.0	106.5	106.1	107.3	105.8
		F = 0.47, p = 0.62			F = 0.18, p =0.83			F = 0.14, p =0.87			F = 1.1, p =0.35			F = 0.48, p =0.62			F = 0.79, p =0.45		
Delayed recall	110.3 ± 12.7	105.8	111.1	110.3	107.9	111.8	110.1	112.4	109.7	110.7	110.7	111.0	108.7	109.5	110.3	110.6	111.0	110.0	109.0
		F = 0.38, p =0.69			F = 0.48, p =0.62			F = 1.3, p =0.27			F = 0.44, p =0.65			F = 0.87, p =0.42			F = 0.71, p =0.49		

P values of co-dominant model of ANCOVA controlling for education years and sex are shown. P values in boldface and underlined mean nominally significant (p < 0.05) and significant for Bonferroni correction for 30 tests (p < 0.0017 =0.05/30), respectively.

**Figure 2:**
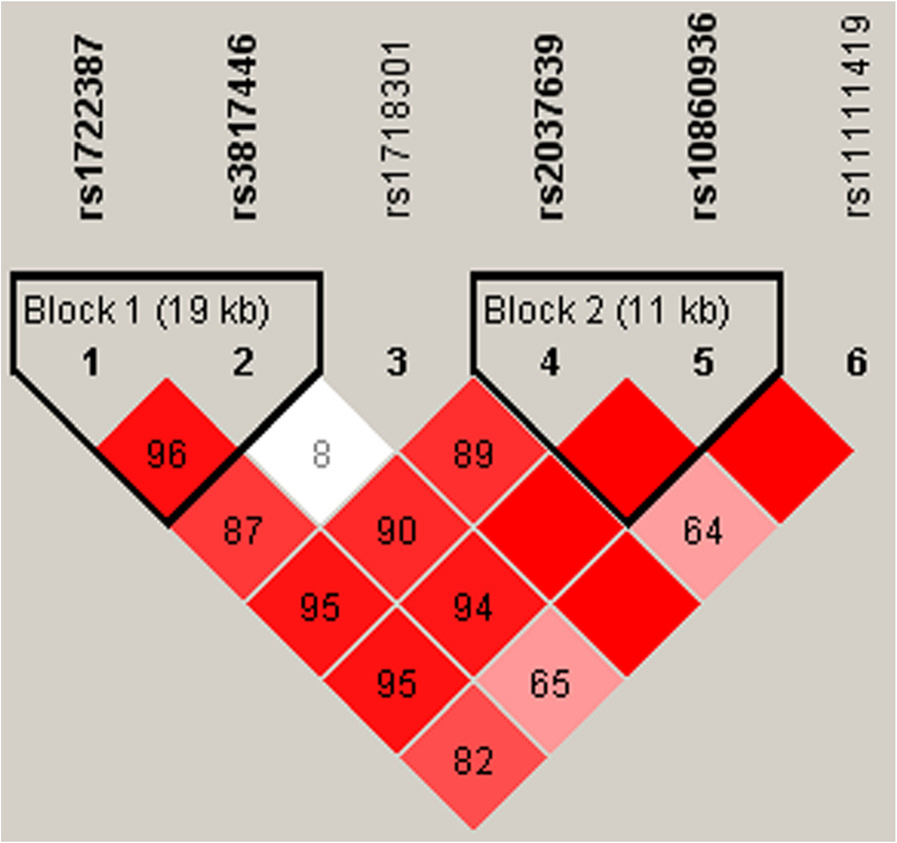
**Haplotype block structure of the *PAH *gene.** Two haplotype blocks (bolded) were constructed for the 6 SNPs of *PAH*, using the Haploview program. Numbers in squares represent the 100 × D‘ values. The blank squares mean D′ = 1.0.

Figure 2 represents LD coefficients (D’) and constructed two haplotype blocks, block 1 (rs1722387 and rs3817446) and block 2 (rs2037639 and rs10860936), for the 6 SNPs of *PAH* obtained by the Haploview software. Haplotype analyses for the constructed two haplotype blocks (blocks 1 and 2) using the PLINK software revealed that haplotype block 2, which contained two markers (rs2037639-rs10860936), was significantly associated with verbal memory index (permutation global p = 0.0091, Table 3).

**Table 3:** **Association of *PAH *haplotypes on verbal memory index of the WMS-R**

	**Haplotype**	**Frequency**	**Adjusted**	**Permutation**
			**p value **^**a**^	**global**
				**p value **^**b**^
Block 1	C-C	0.753	0.060	
(rs1722387- rs3817446)	T-T	0.140	0.12	0.13
	C-T	0.103	0.55	
Block 2	A-T	0.658	0.0068	
(rs2037639-rs10860936)	G-T	0.220	0.25	0.0091
	G-C	0.122	0.012	

^a^ Sex- and education years-adjusted p values for haplotypes of each vs. all others.

^b^ Empirical p values on the basis of 10,000 permutations.

*PAH* phenylalanine hydroxylase gene, *WMS-R* Wechsler Memory Scale-Revised.

## Discussion

We examined whether common genetic variations of *PAH* are associated with memory performance in Japanese healthy adults. To our knowledge, this is the first attempt to examine such an association. We found a nominally significant association of verbal memory index of WMS-R with 5 SNPs (rs1722387, rs3817446, rs2037639, rs10860936 and rs11111419) of the 6 tag SNPs in *PAH.* The association between SNP rs2037639 and verbal memory remained significant after correction for multiple testing. Haplotype-based analysis revealed that a haplotype containing two markers (rs2037639-rs10860936) was significantly associated with verbal memory index. These results suggest that common functional variants in *PAH* impact verbal memory performance in healthy adults. Alternatively, there might be a possibility that functional variants in other genes nearby, which would be in linkage disequilibrium with rs2037639 or the haplotype, may play a role. Since there were no PKU patients in our sample, the obtained result cannot be ascribed to mutations responsible for PKU. Further, considering the carrier frequency for PKU of about 1/150 that corresponds to the PKU incidence among Japanese of 1/70000 to 1/ 120000 [21,22], the number of individuals heterozygous for a rare mutation responsible for PKU is estimated to be less than 5 in our sample. Therefore, it is unlikely that the obtained results are attributable largely to such rare mutations.

Of note, the SNP rs2037639 was previously reported to be associated with schizophrenia in a male Bulgarian sample (nominal p = 0.03) [7]. Since verbal memory performance is one of the most disturbed neurocognitive functions in schizophrenia, it is possible that the SNP confer susceptibility to schizophrenia, at least in part, via affecting memory performance, an endophenotype of the disease.

There is evidence that rare PKU mutations in *PAH* are related with impaired working memory in humans [23,24] and chemically induced (ethylnitrosourea, ENU) mouse model of PKU (*Pah*^enu2^) [25]. However, to our knowledge, there is no report focusing the relationship between common polymorphisms of *PAH* and cognition. Since there is no direct evidence of functional effects of the SNPs, the mechanism underlying the relationship is currently unclear. The SNP (rs2037639) may have some functional effects such as regulation of transcription of *PAH*. Alternatively, the SNP might be in linkage disequilibrium with an unknown functional polymorphism. The functional effect could alter phenylalanine level, which then alters tyrosine level and activation of tyrosine hydroxylase. Finally the *PAH* genotypes could affect dopamine level in the brain [26,27], which is related with memory [28]. In addition, neurotransmitter serotonin was also reportedly found to be decreased in PKU [29-31], possibly being accounted for by inhibitory effect of phenylalanine on the activity of tryptophan hydroxylase [27]. Serotonin also affects dopamine metabolism and serotonin itself is related with human neurocognition.

Previous genetic studies have shown an association of neurocognition with genes encoding enzymes regulating catecholamine metabolism, such as *catechol-O-methyltransferase* (*COMT)* and *monoamine oxidase* (*MAO)*. Krach *et al.* reported that the functional *COMT* Val158Met polymorphism links neural activation pattern during episodic memory tasks [32]. In their study, 84 healthy subjects performed a memory encoding and a retrieval task while they underwent fMRI scanning. Bilateral insula and anterior hippocampus activations were increased linearly with the number of Met alleles (Val/Val [low PFC dopamine] < Val/Met < Met/Met [high PFC dopamine]) during memory encoding. Giakoumaki *et al*. found that tolcapone, the COMT inhibitor, enhanced working memory assessed by the letter–number sequencing tasks in healthy subjects with the Val/Val genotype [33]. On the other hand, Enge *et al*. found that the *MAOA* variable number of tandem repeats (VNTR) polymorphism, which is located in its promoter region and controls the transcriptional activity of the gene, was associated with working memory-related performance, indicating that n-back performance measured by reaction time was poorer in individuals with *MAO-L* (the short allelic variant and lower enzymatic activity) than in individuals with *MAO-H* (the long allelic variant and higher enzymatic activity) [34]. Since PAH converts phenylalanine to tyrosine which is a precursor of dopamine and other catecholamines, functional *PAH* variants could affect synthesis of catecholamines.

There are limitations in the study. The present sample size may have been small, which is subject to type II errors. When we performed a *post-hoc* power analysis to evaluate the statistical power for the association analysis between verbal memory index and the *PAH* rs2037639 genotypes, the current sample size had a 80% power (β = 0.2) to detect mean difference of 3.3, 4.9, and 5.4 between AA vs. AG, AA vs. GG, and AG vs. GG, respectively, at the α (two-tailed) = 5% level. Further studies with a larger sample size might be required to detect smaller differences. The mechanism by which polymorphisms of *PAH* influences memory performance is currently unclear, i.e., there is no direct evidence of functional effects of the examined SNPs. Future animal and human studies are therefore warranted to elucidate the mechanism.

## Conclusions

We performed an association study between genetic variations of *PAH* and memory performance assessed with WMS-R in Japanese healthy adults. We obtained evidence suggesting that genetic variation of *PAH* (SNP rs2037639 and the haplotype containing two markers, rs2037639 and rs10860936) are associated with verbal memory. Considering the carrier frequency for rare mutations responsible for PKU, our data suggest that common functional polymorphisms in *PAH* impact verbal memory. Alternatively, functional polymorphisms in other genes nearby may play a role in genetic basis for verbal memory.

## Abbreviations

ANOVA: Analysis of variance; ANCOVA: Analysis of covariance; COMT: Catechol-O-methyltransferase; DSM- IV: Diagnostic and statistical manual of mental disorders, 4th edition; HWE: Hardy-Weinberg equilibrium; LD: Linkage disequilibrium; MAF: Minor allele frequency; MAO: Monoamine oxidase; MINI: Mini international neuropsychiatric interview; PAH: Phenylalanine hydroxylase; PKU: Phenylketonuria; SNP: Single nucleotide polymorphism; WMS-R: Wechsler memory scale-revised.

## Competing interests

The authors declare that they have no competing interests.

## Authors’ contributions

TT and HK designed the study and TT wrote the draft of the manuscript. TT, DS, HH, KH, MO and SF performed psychiatric assessments for the participants. JM, AN and YK administered the WMS-R. TT and NY performed the genotyping. TF supervised the writing of the paper. HK supervised the project and gave critical comments on the manuscript. All authors contributed to and have approved the final manuscript.

## Supplementary Material

Additional file 1: Table S1 Demographic characteristics. Click here for file
