# Supplementary material for: Possible association between common variants of the phenylalanine hydroxylase (PAH) gene and memory performance in healthy adults
Source: Behav Brain Funct. 2013 Jul 30;9:30. doi: 10.1186/1744-9081-9-30 (PMC3733818; doi:10.1186/1744-9081-9-30)
Supplement: Additional file 1: Table S1 — Demographic characteristics. [file 1744-9081-9-30-S1.doc]

**Table S1 Demographic characteristics.**

Difference between genotype groups was analyzed by analysis of variance (ANOVA) or χ^2^ test. P value in boldface means nominally significant (p < 0.05).

|  | Mean ± S.D. | Single nucleotide polymorphism of *PAH* gene | | | | | | | | | | | | | | | | | |
| --- | --- | --- | --- | --- | --- | --- | --- | --- | --- | --- | --- | --- | --- | --- | --- | --- | --- | --- | --- |
|  |  | rs1722387 | | | rs3817446 | | | rs1718301 | | | rs2037639 | | | rs10860936 | | | rs11111419 | | |
| genotype |  | A/A | A/G | G/G | A/A | A/G | G/G | A/A | A/G | G/G | A/A | A/G | G/G | C/C | C/T | T/T | A/A | A/T | T/T |
| age | 43.8 ± 15.5 | 39.6 | 43.1 | 43.6 | 46.5 | 42.8 | 43.3 | 40.3 | 45.0 | 42.6 | 43.3 | 42.4 | 46.1 | 37.7 | 43.2 | 43.5 | 43.0 | 43.5 | 43.4 |
|  |  | F=1.4, p =0.26 | | | F=0.28, p =0.76 | | | F=2.9, p =0.058 | | | F=1.3, p =0.27 | | | F=1.4, p=0.26 | | | F=0.52, p =0.60 | | |
| education years | 15.2 ± 2.7 | 15.6 | 15.4 | 15.2 | 15.2 | 15.6 | 15.2 | 15.1 | 15.3 | 15.4 | 15.2 | 15.6 | 14.7 | 16.1 | 15.4 | 15.2 | 15.3 | 15.3 | 15.4 |
|  |  | F=0.37, p =0.69 | | | F=0.96, p=0.38 | | | F=0.44, p =0.64 | | | F=3.2, **p=0.04** | | | F=0.46, p =0.63 | | | F=0.71, p =0.49 | | |
| smoking status | n=113  (19.0  %smokers) | 2 | 32 | 79 | 7 | 40 | 66 | 5 | 28 | 80 | 46 | 53 | 13 | 2 | 26 | 84 | 70 | 35 | 7 |
|  |  | χ^2^=2.4, p=0.31 | | | χ^2^=0.22, p=0.90 | | | χ^2^=3.1, p=0.21 | | | χ^2^=1.2, p=0.55 | | | χ^2^=0.58, p =0.75 | | | χ^2^=0.43, p=0.81 | | |

*PAH*, phenylalanine hydroxylase
